# Clinical Outcomes of Specific Immunotherapy in Advanced Pancreatic Cancer: A Systematic Review and Meta-Analysis

**DOI:** 10.1155/2017/8282391

**Published:** 2017-02-07

**Authors:** Jiang Chen, Guo Xiao-Zhong, Xing-Shun Qi

**Affiliations:** Department of Gastroenterology, Shenyang General Hospital of PLA, No. 83 Wenhua Road Shenyang City, Liaoning 110016, China

## Abstract

Specific immunotherapies, including vaccines with autologous tumor cells and tumor antigen-specific monoclonal antibodies, are important treatments for PC patients. To evaluate the clinical outcomes of PC-specific immunotherapy, we performed a systematic review and meta-analysis of the relevant published clinical trials. The effects of specific immunotherapy were compared with those of nonspecific immunotherapy and the meta-analysis was executed with results regarding the overall survival (OS), immune responses data, and serum cancer markers data. The pooled analysis was performed by using the random-effects model. We found that significantly improved OS was noted for PC patients utilizing specific immunotherapy and an improved immune response was also observed. In conclusion, specific immunotherapy was superior in prolonging the survival time and enhancing immunological responses in PC patients.

## 1. Introduction

Pancreatic cancer (PC) is a fatal disease with high mortality and poor prognosis. In the United States, PC is the fourth leading cause of cancer-related deaths, and it resulted in the death of 40,560 Americans in 2015 [1]. Pancreatic adenocarcinoma, which is derived from the glandular tissue of the pancreas, forms the majority of PC [2]. The median overall survival (MOS) time is 4–6 months in patients with metastatic disease, and the 5-year survival rate of patients following R0 pancreatic surgery is less than 20% [3]. The symptoms of PC typically occur late; as such, patients are diagnosed in advanced stages. The high mortality rate of patients with PC can partially be attributed to the lack of effective therapies. Current therapeutic options for PC are limited to surgical resection, systemic chemotherapy, and radiotherapy, but none of these strategies can completely treat this condition [4]. Therefore, effective treatment methods should be developed.

Immunotherapy is a promising treatment option considered as the fourth most common therapeutic method for cancer [5]. In cancer immunotherapy, the immune system is employed to reject tumors and to prevent recurrence. Cancer immunotherapy comprises passive, active, or immunomodulatory approaches. Passive immunotherapy involves the administration of exogenously generated antibodies or adoptively transferred immune cells, typically T cells, to mediate an anticancer immune response. Immunomodulatory agents enhance immune responses to improve the immunity to cancer. In active immunotherapy, endogenous immune cells are activated to recognize specific tumor-associated antigens (TAAs) and eliminate cancer cells with minimal damage to healthy nontumor cells. Furthermore, cancer immunotherapy can be divided into nonspecific and specific immunotherapy on the basis of specific tumor antigens.

Conventional strategies used to treat PC include nonspecific immunotherapies, such as exogenous immunostimulants, cytokines, and adoptive transfer of nonspecific immune effector cells. Another strategy involves the inhibition of negative immune regulatory pathways and tumor-derived immune suppressive molecules. Clinical results have been evaluated to nonspecific immunotherapies in patients with PC, but the response rate, progression-free survival, or overall survival has yet to be improved [6, 7]. In general, nonspecific approaches have yielded limited results regarding the treatment of PC.

In specific immunotherapy, vaccines with autologous tumor cells and tumor antigen-specific monoclonal antibodies are used. This technique elicits a long-term antitumor immune response and thus is more effective than other approaches in a minimal residual disease setting [33]. Since the discovery of TAAs in the early 1990s, identification of antigens and description of immune interactions in cancer patients have been enhanced. Clinical trials have been conducted on specific immunotherapy for PC by using autologous tumor cell vaccines, defined tumor protein vaccines, monoclonal antibody and anti-idiotypic vaccines, multipeptide vaccines, viral vector vaccines, naked DNA vaccines, and dendritic cell (DC) vaccines [33].

Despite the abundance of preclinical data, the efficacy of specific immunotherapy against PC has been rarely described. Early clinical trials on specific immunotherapy against PC have provided mixed results, which cause controversial insights into the clinical efficacy of specific immunotherapy against PC. In this study, the potential beneficial effects of specific immunotherapy on PC were investigated and the clinical outcomes of specific immunotherapy were evaluated on the basis of the survival, immune system function, and tumor markers of patients with PC.

## 2. Methods

### 2.1. Search Strategy and Selection Criteria

The PubMed, EMBASE, Cochrane Library, and China Science and Technology Journal Databases were searched for the relevant publications. The following search terms were used: “specific immunotherapy” or “immunotherapy” or “immunologic adjuvant” or “vaccine” or “vaccination” or “autologous tumor cell” or “dendritic cell” and “pancreatic cancer” or “pancreatic adenocarcinoma”. An initial search was performed on November 13, 2015, and updated searches were conducted on August 1, 2016. Manual searches of reference lists, conference proceedings of the American Society of Clinical Oncology Annual Meetings, and the European Cancer Conference were carried out. https://www.ClinicalTrials.gov website was also searched for information on prospective and ongoing trials.

Eligibility criteria were as follows: (a) the publications were human clinical studies but not reviews, comments, letters, or basic science research; (b) the sample size was ≥ 6; (c) the participants were diagnosed with advanced PC without any other kinds of malignant tumor; (d) the participants received cancer-specific immunotherapy; (e) the publication language was not limited; (f) no concurrent chemotherapy, radiotherapy, or drugs which affect immune function (such as glucocorticoids and cimetidine) were administered during cancer-specific immunotherapy or follow-up; (g) the routes of cancer-specific immunotherapy were not restricted; (h) if the data overlapped or were duplicated among two or more studies by the same study team, only the study with more complete data or one earlier study was included; and (i) full text data without appropriate control arm or abstracts that were never subsequently published as full papers were excluded. Moreover, publication years or study design was not restricted.

### 2.2. Data Extraction

Two reviewers independently selected the trials and performed the data extraction. Discrepancies were resolved by discussion among reviewers. Primary items were extracted as follows: first author, publication journal, publication year, regions of study, demographic data (age and gender), number of patients and number of patients analyzed, study design, data enrollment period, stage of disease, cancer-specific immunotherapy arm and control arm design, type and dose of specific immunotherapy administered, length of follow-up, and procedure-related complications.

### 2.3. Quality Assessment

The quality of the study was assessed on the basis of the type of comparison.

For studies with self-controlled data, the following questions were evaluated:

1. Were the patients prospectively enrolled?
2. Was the enrollment period reported?
3. Were the eligibility criteria reported?
4. Were the demographic data (gender and age) reported?
5. Was the tumor stage of PC reported?
6. Was the detailed course of specific immunotherapy reported?
7. Was the length of follow-up reported?

For studies with case-control data, the following questions were evaluated:

1. Were the patients prospectively enrolled?
2. Were the patients randomly assigned to treatment or control groups?
3. Were the randomization methods clearly described?
4. Was the treatment modality of the control group clearly described?
5. Were the eligibility criteria reported?
6. Were the demographic data (gender and age) similar between treatment and control groups?
7. Were the tumor stages of PC similar in the patients in treatment and control groups?

If one study had ≥6 answers with “Yes,” then the study was considered high quality; otherwise, the study was considered low quality.

### 2.4. Definition of Clinical Outcomes

The following clinical outcomes were considered to evaluate specific immunotherapy in advanced PC: overall survival (OS), immune response data, and serum cancer marker data.

OS was defined as the time from the initiation of treatment until death from any case. Serum cancer marker data, including cancer embryonic antigen (CEA) and carbohydrate antigen (CA) 19-9, prompted the nature of the tumor before and after cancer-specific immunotherapy. The immune response was assessed by evaluating and comparing the data of antibody and cytotoxic T lymphocytes (CTL) and levels of immunocytokines, such as IFN-*γ* and IL-4, from the included papers. The data of CD4^+^, CD8^+^, CD56^+^, CD4^+^/CD8^+^, and CD4^+^CD25^+^ cell populations were extracted from the recruited papers.

### 2.5. Statistical Analysis

Data were analyzed using Review Manager Version 5.3 (Nordic Cochran Center, Copenhagen, Denmark). In our meta-analysis, the immunotherapy-containing arms of the identified trails were compared with the respective nonimmunotherapy arms. For dichotomous variables, pooled odds ratios (OR) with 95% confidence intervals (95% CI) were calculated to assess treatment efficacy. For continuous variables, the mean difference (MD) with 95% CI was calculated from the included studies. *P* < 0.05 was considered statistically significant for the effect size. Data were pooled with a random-effects model. The heterogeneity among the studies was assessed by *I*^2^ statistic (*I*^2^ > 50% indicated substantial heterogeneity) and Chi-square test (*P* < 0.10 was considered to represent significant statistical heterogeneity). Egger's test was implemented to analyze the publication biases. Subgroup analyses were conducted according to the type of specific immunotherapy (active specific immunotherapy/positive specific immunotherapy), tumor stage (grade III and IV/grade IV alone/mixed or unknown), study quality (high/low), and continent where the study was conducted (America/Asia/Europe).

## 3. Results

### 3.1. Study Selection

**Figure 1:**
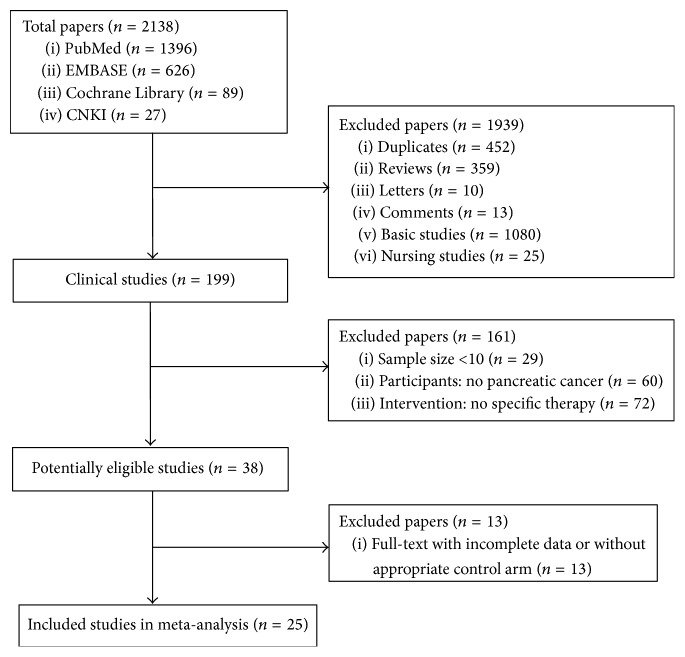
Flowchart of inclusion.

A total of 2138 papers were identified from the four databases, and, among them, 2100 publications were excluded for various reasons (452 were duplicates, 359 were review articles, 23 were letters and comments, 1080 were basic studies, 25 were nursing studies, 29 were with sample size < 6, 60 had no pancreatic cancer, and 72 were without specific immunotherapy). A total of 38 clinical trials were selected as potentially relevant, and their full texts were retrieved for a more detailed assessment. Of the 38 studies, 13 were excluded because they did not provide detailed patient clinical data or a control arm and therapy response. The procedure used to select the clinical trials is shown in Figure 1. As a result, 25 articles reporting clinical trials of active specific immunotherapy were selected for the meta-analysis [8–32].

### 3.2. Characteristics of the Included Studies

The characteristics of the 25 papers are shown in Table 1. The countries included Japan (*n* = 9) [8, 13, 15–17, 24, 28, 30, 31], China (*n* = 6) [11, 21, 22, 26, 29, 32], UK (*n* = 3) [10, 23, 27], Germany (*n* = 1) [18], Norway (*n* = 2) [9, 14], and USA (*n* = 4) [12, 19, 20, 25].

The total number of patients analyzed in the included studies was 1908. The number of patients varied from 6 to 1062, and half of the patients were male. With respect to the age of patients, all 25 studies included adult patients (age > 30 years). With respect to the grade of tumor stage, 4 studies included patients with WHO grade IV alone, 5 included patients with WHO grades III and IV, and 16 included patients with mixed or unknown tumor stage grades.

Of the 25 studies, 12 reported the self-controlled data before and after cancer-specific immunotherapy. In addition, 13 studies reported the case-controlled data between the treatment and control groups, of which 6 and 7 were case-controlled and randomized controlled studies, respectively. The types of the cancer-specific immunotherapy included tumor antigen peptide vaccine (*n* = 9) [8, 9, 14, 15, 23, 24, 28, 30, 31], DC pulsed by cancer antigens (*n* = 6) [11, 13, 16, 17, 26, 31], monoclonal antibody (*n* = 6) [10, 12, 19, 20, 25, 27], DC-CIK (*n* = 3) [21, 29, 32], and lymphoblastoid cell lines (*n* = 1) [18]. The length of follow-up of 25 trials ranged from 7 days to more than 3 years.

### 3.3. Study Quality

For the self-controlled data, 23 and 8 papers were considered to be of high and low quality, respectively (see Supplementary Table  1 in Supplementary Material available online at https://doi.org/10.1155/2017/8282391). With regard to the case-control data, 6 and 7 papers were regarded as high and low quality, respectively* (Supplementary Table  2)*.

### 3.4. Procedure-Related Complication

Procedure-related complications included nausea, fever, pain at the bone marrow puncture points, and hematoma at the femoral artery puncture points. These complications were mild and thus subsided spontaneously. Moreover, no severe procedure-related complications were reported. 

### 3.5. Overall Survival

#### 3.5.1. 3-, 6-, and 12-Month OS

**Figure 2:**
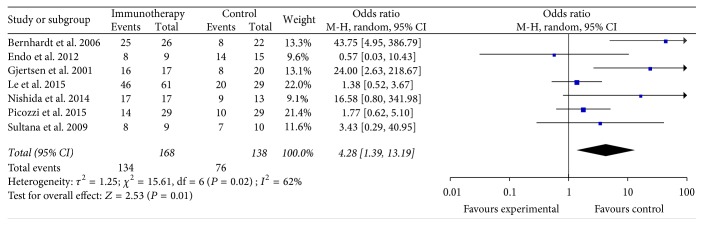
Forest plot of comparison: 3-month overall survival of 7 included studies.

The data of the 3-month OS were available in seven studies [9, 13, 14, 20, 24, 25, 27]. These seven studies contained 306 patients (168 patients received specific immunotherapy and 138 control patients did not receive specific immunotherapy). The lengths of the follow-up periods are summarized in Table 1. The 3-month OS of the PC patients who received specific immunotherapy was significantly higher than that of the nonspecific immunotherapy group (OR: 4.28, 95% CI: 1.39–13.19, *P* = 0.01) (Figure 2). The heterogeneity among the studies was statistically significant (*P* = 0.02, *I*^2^ = 62%), but the publication bias was not statistically significant (Egger: bias = 2.01, 95% CI: −1.13 to 5.16, *P* = 0.16).

**Figure 3:**
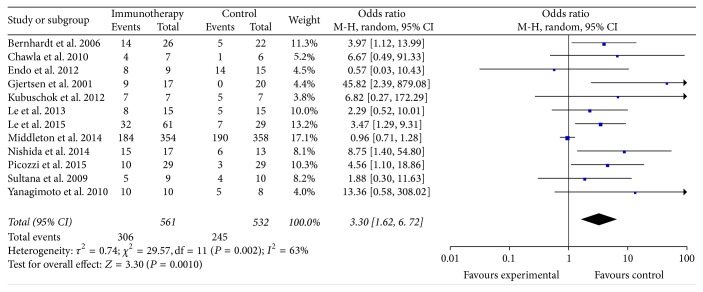
Forest plot of comparison: 6-month overall survival (12 studies).

The data of the 6-month OS were available for 12 trials [9, 12–14, 18–20, 23–25, 27, 30]. These 12 trials included 1093 patients (561 patients received specific immunotherapy and 532 control patients did not receive specific immunotherapy). The results showed that the 6-month OS of the PC patients who received specific immunotherapy was significantly higher than that of the nonspecific immunotherapy group (OR: 3.30, 95% CI: 1.62 to 6.72, *P* = 0.001) (Figure 3). The heterogeneity among the studies was statistically significant (*P* = 0.002, *I*^2^ = 63%) as was the publication bias (Egger: bias = 1.79, 95% CI: 0.94 to 2.64, *P* = 0.0008).

**Figure 4:**
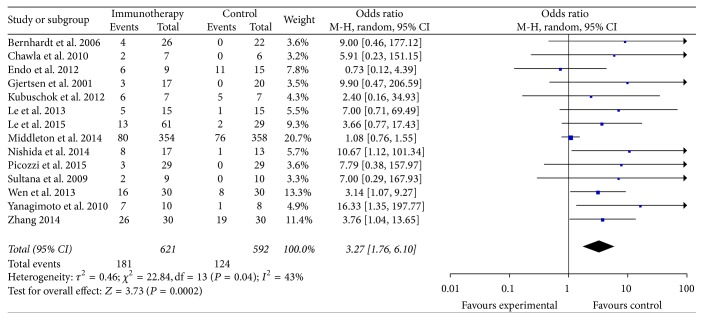
Forest plot of comparison: 1-year overall survival of 14 included studies.

The data of the 1-year OS were available in 14 trials [9, 12–14, 18–20, 23–25, 27, 29, 30, 32] and included 1213 patients (621 patients received specific immunotherapy and 592 control patients did not receive specific immunotherapy). The meta-analysis revealed that the 1-year OS of the patients who received specific immunotherapy was significantly higher than those who did not (OR: 3.27, 95% CI: 1.76 to 6.10, *P* = 0.0002). Cochran's *Q* test yielded *P* = 0.04, and the corresponding *I*^2^ was 43% (Figure 4). The publication bias (Egger: bias = 1.54, 95% CI: 0.95 to 2.14, *P* = 0.0001) was statistically significant.

The results of the subgroup meta-analyses were demonstrated in* Supplementary Table  3.*

#### 3.5.2. 1.5-, 2-, and 3-Year OS

**Figure 5:**
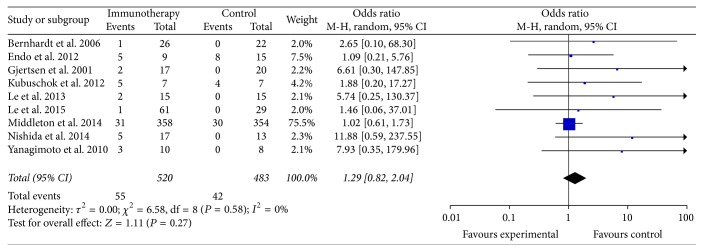
Forest plot of comparison: 1.5-year overall survival of 9 included studies.

Nine studies reported the 1.5-year OS of patients in the specific immunotherapy and control groups [9, 13, 14, 18–20, 23, 24, 30], which included a total of 1003 patients (520 patients received specific immunotherapy). The lengths of the follow-up periods were summarized in Table 1. These nine trials showed that the 1.5-year OS of the PC patients who received specific immunotherapy did not significantly improve compared with that of the nonspecific immunotherapy group (OR: 1.29, 95% CI: 0.82 to 2.04, *P* = 0.27). The heterogeneity among the studies was not statistically significant (*P* = 0.58, *I*^2^ = 0%) (Figure 5). The publication bias was statistically significant (Egger: bias = 1.06, 95% CI: 0.46 to 1.67, *P* = 0.0042).

**Figure 6:**
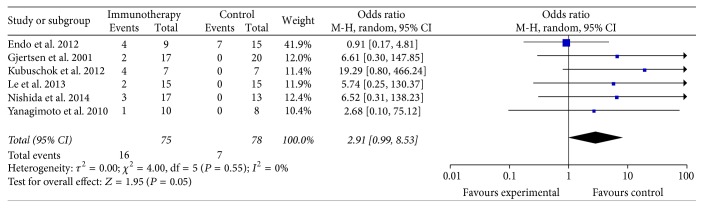
Forest plot of comparison: 2-year overall survival (6 studies).

Six studies reported the 2-year OS of patients in the specific immunotherapy and control groups [13, 14, 18, 19, 24, 31], which included a total of 153 patients (75 patients received specific immunotherapy). These six trials did not show a longer OS among patients who received specific immunotherapy than those who did not, and the estimated pooled OR for these six trials did not reveal a significantly improved 2-year OS among PC patients receiving specific immunotherapy (OR: 2.91, 95% CI: 0.99–8.53, *P* = 0.05) (Figure 6). The heterogeneity among the studies was statistically insignificant (*P* = 0.55, *I*^2^ = 0%), and the publication bias was statistically significant (Egger: bias = 2.55, 95% CI: 0.68 to 4.42, *P* = 0.0192).

**Figure 7:**
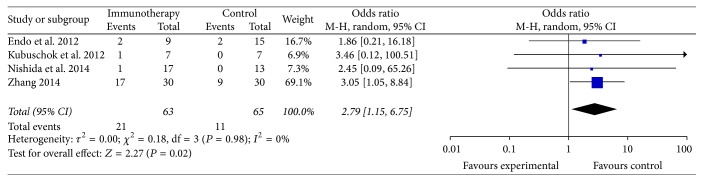
Forest plot of comparison: 3-year overall survival of 4 included studies.

The data of the 3-year OS were available in four trials [13, 18, 24, 32]. The four trials included 128 patients (63 patients received specific immunotherapy and 65 control patients who did not receive specific immunotherapy were used as controls). The meta-analysis showed that the 3-year OS of the patients who received specific immunotherapy was significantly improved compared with that of the patients who did not undergo treatment (OR: 2.79, 95% CI: 1.15–6.75, *P* = 0.02) (Figure 7). The heterogeneity among the studies (*P* = 0.98, *I*^2^ = 0%) and the publication bias (Egger: bias = −0.18, 95% CI: −1.48 to 1.12, *P* = 0.6165) were not statistically significant.

The results of the subgroup meta-analyses were demonstrated in* Supplementary Table  4.*

### 3.6. Immune Response

#### 3.6.1. Comparison of CTL and Antibody-Responses before and after Specific Immunotherapy in PC Patients

**Figure 8:**
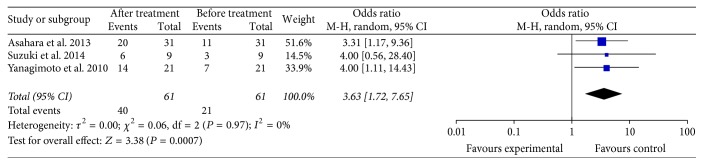
Forest plot of comparison: CTL-responses between before and after specific immunotherapy treatment groups (3 studies).

Three studies reported the CTL response change before and after specific immunotherapy in PC patients [8, 28, 30]. Three trials included 61 patients who received specific immunotherapy. The lengths of the follow-up periods were summarized in Table 1. The meta-analysis showed the CTL response of the patients who received specific immunotherapy (OR: 3.63, 95% CI: 1.72 to 7.65, *P* = 0.0007) was significantly improved (Figure 8). Cochran's *Q* test yielded *P* < 0.97, and the corresponding *I*^2^ was 0%. The publication bias (Egger: bias = 0.41, 95% CI: −3.10 to 3.92, *P* = 0.5037) was not statistically significant.

**Figure 9:**
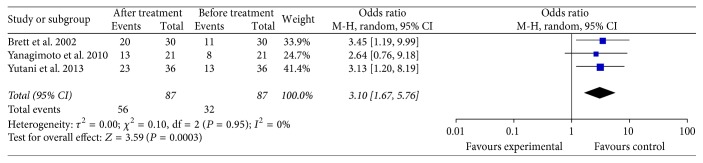
Forest plot of comparison: antibody-responses between before and after specific immunotherapy treatment groups (3 studies).

The data of the antibody-response change were available in three trials [10, 30, 31], which included 87 PC patients who received specific immunotherapy. These three trials showed that the antibody-response of patients who received specific immunotherapy (OR: 3.10, 95% CI: 1.67–5.76, *P* = 0.0003) was significantly improved (Figure 9). Cochran's *Q* test yielded *P* = 0.95, and the corresponding *I*^2^ was 0%. The publication bias statistically insignificant (Egger: bias = −1.17, 95% CI: −10.95 to 8.62, *P* = 0.4968).

The results of the subgroup meta-analyses were demonstrated in* Supplementary Table  5.*

#### 3.6.2. Comparison of Lymphocyte Subsets in the Peripheral Blood of PC Patients

**Figure 10:**
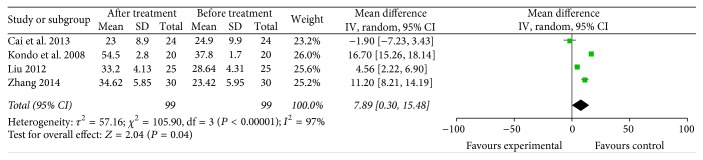
Forest plot of comparison: CD4^+^ lymphocyte subset percentages between specific immunotherapy group and the baseline observed before treatment group in 4 included studies.

**Figure 11:**
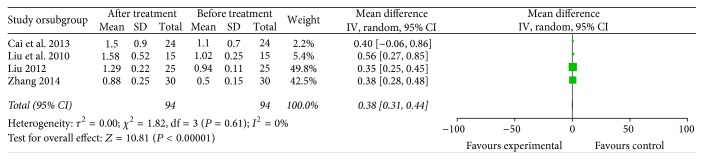
Forest plot of comparison: portion of CD4^+^/CD8^+^ between specific immunotherapy group and the baseline observed before treatment group (4 studies).

The meta-analysis results showed that the proportions of CD4^+^ (4 trials included 99 patients who received specific immunotherapy) [11, 17, 21, 32] and CD4^+^/CD8^+^ (4 trials included 94 patients who received specific immunotherapy) [11, 21, 22, 32] cells were significantly increased after the specific immunotherapy was administered, as indicated by the estimated pooled MD of 7.89 (95% CI: 0.30–15.48, *P* = 0.04) and 0.38 (95% CI: 0.31 to 0.44, *P* < 0.00001). Cochran's *Q* test had *P* < 0.00001 and *P* < 0.00001. The corresponding *I*^2^ were 97% and 0% (Figures 10 and 11). The publication bias (Egger: bias = −10.10, 95% CI: −32.68 to 12.48, *P* = 0.194) and (Egger: bias = 0.95, 95% CI: −2.06 to 3.95, *P* = 0.308) were not statistically significant.

**Figure 12:**
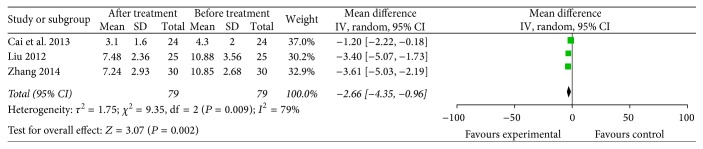
Forest plot of comparison: CD4^+^CD25^+^ lymphocyte subset percentages between specific immunotherapy group and the baseline observed before treatment group (3 studies).

CD4^+^CD25^+^ cells (3 trials included 79 patients who received specific immunotherapy) [11, 21, 32] were significantly lower in the specific immunotherapy group than the baseline observed before treatment, as shown by the pooled MD of −2.66 (95% CI: −4.35 to −0.96, *P* = 0.002). Cochran's *Q* test had *P* = 0.009. The corresponding *I*^2^ was 79% (Figure 12). The publication bias statistically insignificant (Egger: bias = −7.938, 95% CI: −49.56 to 33.68, *P* = 0.249).

**Figure 13:**
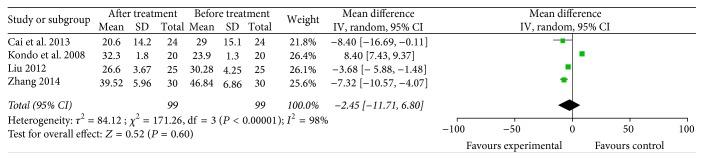
Forest plot of comparison: CD8^+^ lymphocyte subset percentages between specific immunotherapy group and the baseline observed before treatment group in 4 included studies.

**Figure 14:**
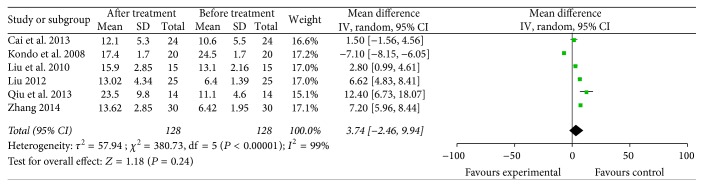
Forest plot of comparison: CD56^+^ lymphocyte subset percentages between specific immunotherapy group and the baseline observed before treatment group in 6 included studies.

CD8^+^ [11, 17, 21, 32] and CD56^+^ [11, 17, 21, 22, 26, 32] lymphocyte subsets (4 trials with 99 patients who received specific immunotherapy and 6 trials with 128 patients who received specific immunotherapy) were not significantly increased after specific immunotherapy treatment compared with the observed baseline, as indicated by the pooled MD of −2.45 (95% CI: −11.71 to 6.80, *P* = 0.60) and 3.74 (95% CI: −2.46 to 9.94, *P* = 0.60). Cochran's *Q* test had *P* < 0.00001, and the corresponding *I*^2^ were 98% and 99% (Figures 13 and 14). The publication bias (Egger: bias = −9.45, 95% CI: −28.30 to 9.39, *P* = 0.164) and (Egger: bias = 8.00, 95% CI: −15.61 to 31.63, *P* = 0.40) were not statistically significant.

The results of the subgroup meta-analyses were demonstrated in* Supplementary Table  6.*

#### 3.6.3. Comparison of Immune Cytokine Levels in the Peripheral Blood of PC Patients

**Figure 15:**
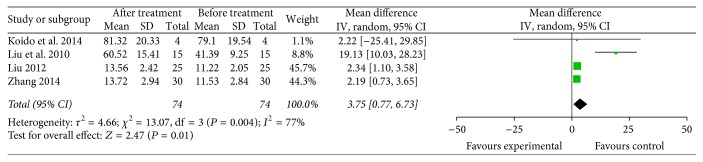
Forest plot of comparison: IFN-*γ* levels between specific immunotherapy group and the baseline observed before treatment group (4 trials).

The meta-analysis showed that the IFN-*γ* level (4 trials with 81 patients who received specific immunotherapy) [16, 21, 22, 32] in the specific immunotherapy group was significantly higher than the corresponding baseline before treatment, as revealed by the pooled MD of 3.75 (95% CI: 0.77 to 6.73, *P* = 0.01). Cochran's *Q* test had *P* = 0.004, and the corresponding *I*^2^ was 77% (Figure 15). The publication bias was statistically insignificant (Egger: bias = 1.857, 95% CI: −4.637 to 8.353, *P* = 0.344).

**Figure 16:**
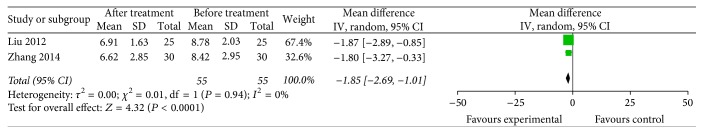
Forest plot of comparison: IL4 levels between specific immunotherapy group and the baseline observed before treatment group (2 trials).

The IL-4 level (2 trials with 55 patients who received specific immunotherapy) [21, 32] was significantly decreased after specific immunotherapy treatment, as indicated by the pooled MD of −1.85 (95% CI: −2.69 to −1.01, *P* < 0.0001). Cochran's *Q* test had *P* = 0.94, and the corresponding *I*^2^ was 0% (Figure 16).

The results of the IFN-*γ* level subgroup meta-analysis were demonstrated in* Supplementary Table  7.* The IL-4 level subgroup meta-analysis was not performed due to the similarity of conclusions among the studies.

### 3.7. Serum Cancer Markers

**Figure 17:**
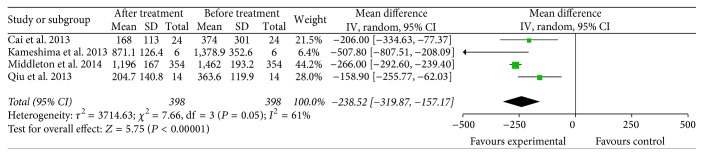
Forest plot of comparison: CA19-9 levels between specific immunotherapy group and the baseline observed before treatment group (4 trials).

Four studies (with 398 patients who received specific immunotherapy) reported a decrease in the serum cancer marker CA19-9 of the specific immunotherapy group compared with the corresponding baseline before treatment [11, 15, 23, 26]. The results of the meta-analysis showed a significant decrease in the CA19-9 levels between the two groups with pooled MD of −238.52 (95% CI: −319.87 to −157.17, *P* < 0.00001). Cochran's *Q* test had *P* = 0.05. The corresponding *I*^2^ was 61% (Figure 17). The publication bias (Egger: bias = 0.332, 95% CI: −5.84 to 6.50, *P* = 0.838) was not statistically significant.

**Figure 18:**
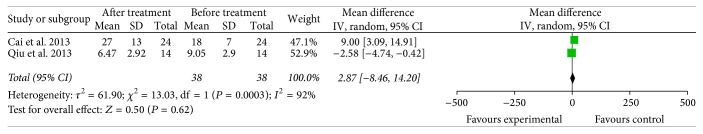
Forest plot of comparison: CEA levels between specific immunotherapy group and the baseline observed before treatment group (2 trials).

CEA was another selected serum cancer marker, and two trials provided sufficient data (38 patients who received specific immunotherapy) [11, 26] for meta-analysis. The CEA level was not significantly decreased in the specific immunotherapy group compared with the corresponding baseline before treatment, as shown by the pooled MD value of 2.87 (95% CI: −8.46 to 14.20, *P* = 0.62). Cochran's *Q* test had *P* = 0.0003, and the corresponding *I*^2^ was 92% (Figure 18).

The results of the CA19-9 level subgroup meta-analysis were demonstrated in* Supplementary Table  8.* The CEA level subgroup meta-analysis was not performed due to the similarity of conclusions among the studies.

## 4. Discussion

With a 5-year survival rate of 8%, PC is projected to be the second leading cause of cancer deaths by 2030 [34]. Traditional treatments for PC are also limited and ineffective. Therefore, more efficacious therapies should be identified and developed. As a promising alternative, immunotherapy is widely considered the fourth-line treatment modality for patients with cancer [35, 36]. Since varying evidence showing that PC can elicit antitumor immune responses was initially reported, the use of specific immunotherapy for the treatment of PC has caused a worldwide concern [37, 38]. However, specific immunotherapy can provide encouraging results in preclinical models but often fail to show clear benefits in clinical trials for PC [39, 40]. Our study is the first systematic review and meta-analysis that examines the clinical efficacy of different PC-specific immunotherapy strategies, including active specific immunotherapy (ASI) (e.g., therapeutic vaccines or immunomodulatory agents that eventually lead to the expansion of tumor-specific T cells) and passive specific immunotherapy (PSI) (e.g., tumor-specific immune effector cells or antibodies that mediate an immune response) by collecting currently available evidence.

Our meta-analysis obtained several remarkable findings. An important finding was that the specific immunotherapy could significantly improve the 3-, 6-, and 12-month OS (*P* < 0.05) of PC patients compared with the nonspecific immunotherapy control groups. The advantage of logistic regression was evident, as shown in Figure 2. For longer-term survival, our analyses found that the specific immunotherapy was associated with a significantly prolonged 3-year OS of PC patients (*P* < 0.05), but no effects on 1.5- and 2-year OS (*P* ≥ 0.05) were observed. This phenomenon might occur because the majority of patients were in III or IV stages. The prolongation of longer-term survival in PC patients was limited for the specific immunotherapy. This finding suggested that improving diagnostic methods and fulfilling a major shift of PC from stages III or IV to stages II or I were necessary to increase the number of PC patients who will procure benefits with PC-specific immunotherapy. Another reason could be that 1.5- and 2-year OS subgroups included 1276 PC patients in a total of 15 trials when we collected the clinical data. By contrast, 3-year OS subgroups included only 160 patients in 4 trials. Therefore, the total sample size of the 3-year OS subgroup was insufficient compared with the 1.5- and 2-year OS subgroups, and these findings affected the results of our meta-analysis. These findings were similar to those described by Chen and Zhang [41].

Another important finding was that specific immunotherapy could significantly upregulate the immune response of patients with PC, including an increase in tumor antigen-specific CTL response (*P* < 0.05) and tumor antigen-specific antibody-response (*P* < 0.05). Numerous data have shown how PC patients generate B and T cells specific to antigens expressed on autologous pancreatic tumor cells in support of the PC-specific immunotherapy approaches [42, 43]. This finding suggested that the therapeutic efficacy of specific immunotherapies is generally correlated with the generation of strong antigen-specific T- and B-cell responses, and the enhancement of such responses may increase the overall potency of specific immunotherapies.

Lymphocytes play a crucial role in tumor cell eradication, and human immune responses against a tumor are mainly dependent on cellular immunity. The ratios of T-lymphocyte subsets in the peripheral blood are usually distorted in tumor patients [44]. The specific immunotherapies may be used to regulate the percentages of lymphocyte cells in PC patients. CD4^+^ T cells are also necessary to enhance host antitumor responses and CD8^+^ T-lymphocyte priming [45]. The ratio of CD4^+^/CD8^+^ cells is related to the status of the immune system. In the present analysis, the percentages of CD4^+^ T cells and CD4^+^/CD8^+^ cells were significantly increased in the specific immunotherapy group compared with corresponding baseline before treatment (*P* < 0.05). These data implied that the specific immunotherapy can enhance cellular immune function and potent systemic antitumor activity. The presence of tumor-specific CD8^+^ T cells (CTL) in the peripheral blood plays an important role in recognizing target antigens and lysing tumor cells by direct contact. The percentages of CD8^+^ T cells in patients who receive specific immunotherapy may be increased [46]. However, we did not find any significant difference in the percentages of CD8^+^ T cells between the two groups after specific immunotherapy treatment (*P* = 0.60). The potential reason might be that the majority of the patients included in this meta-analysis subgroup were treated with chemotherapy. Compared with the other subsets of T lymphocytes, the CD8^+^ T cells are more sensitive to cytotoxic chemotherapy and no less than 3 months are required after chemotherapy is terminated to return to baseline cell numbers regardless of the patient's age [47]. Kondo et al. [17] confirmed this finding in the same subgroup. The CD8^+^ cells in the patients who did not receive the chemotherapy significantly increased after specific immunotherapy was administered. However, combining specific immunotherapy with chemotherapy can recognize and kill cancer cells and help increase the sensitivity of tumor cells to chemotherapy compared with specific immunotherapy alone [48]. Thus, combination therapy is more effective than single therapy.

CD56^+^ T cells are natural killing (NK) cells that participate in the nonspecific immune eradication of tumor cells in vivo. The analysis of CD56^+^ T cells demonstrated that NK cell (*P* = 0.24) percentages did not differ between the specific immunotherapy group and the corresponding baseline group before treatment. The findings suggested that the effects of nonspecific immune responses on patients of PC might not be associated with the specific immunotherapy. However, the changes in the NK cell percentage might not have been observed because of a short follow-up time in most of the studies included.

T helper (Th) 1 and Th2 cells are two important T regulatory (Treg) (CD4^+^CD25^+^) cells in the body. Treg cells transferring from Th1 to Th2 are a phenomenon unique to malignant tumor. The development of Th2 cells will promote the long-term retention of cancer cells in the host body and protect from immune surveillance and immune attack. Th1 and Th2 cells costimulate IFN-*γ* production, whereas Th2 cells preferentially induce more IL-4 production than Th1 cells do [49]. In our meta-analysis, the IFN-*γ* levels in the peripheral blood of PC patients significantly increased after specific immunotherapy treatment (*P* < 0.05). By contrast, the IL-4 levels significantly decreased in the specific immunotherapy group compared with the baseline levels before treatment (*P* < 0.001). These results were fitted with our other finding obtained from the same meta-analysis showing that the percentages of CD4^+^CD25^+^ Treg cells were significantly decreased in the specific immunotherapy group compared with the corresponding baseline group before treatment (*P* < 0.05). These results suggested that affecting the Th1/Th2 cytokine network and decreasing the portion of Treg cells might be a potential mechanism for the specific immunotherapy treatment in the patients of PC. Several strategies targeting Tregs in vivo have been employed with certain efficacy in cancer, including depletion with anti-CD25 antibodies and treatment with anti-GITR and anti-CTLA-4 [50, 51]. These strategies are called immune checkpoint therapy. Royal et al. [40] investigated the role of single agent ipilimumab, an anti-CTLA-4 antibody, in a cohort of locally advanced or metastatic pancreatic adenocarcinoma. In this phase 2 trial, a significant delayed regression of metastatic PC is observed in 1 out of 27 patients enrolled in the study. The findings were particularly interesting because they demonstrated that the mechanism of action of ipilimumab involves immunomodulation rather than direct tumoricidal activity.

The third important finding was that the levels of CA19-9 significantly decreased in the specific immunotherapy group compared with the corresponding baseline before treatment (*P* < 0.05). Moreover, CEA levels did not significantly decrease after specific immunotherapy treatment (*P* = 0.62). PC patients with increased serum CEA and CA19-9 levels at diagnosis demonstrate poor OS, and pretreatment CEA and CA19-9 levels may predict the prognosis of patients with PC [52]. The results of our meta-analysis suggested that the specific immunotherapy could inhibit the tumor progression and effectively improve the prognosis in PC patients. The CEA levels did not significantly decrease after specific immunotherapy treatment, and this observation might be explained by the insufficient number of patients enrolled in this analysis.

This study has several strengths. First, an extensive search of the relevant studies was conducted via four major databases, and the publication language was not restricted. Second, given the potential heterogeneity among the studies, only a random-effects model was employed to obtain a conservative result. Third, the eligibility criteria were strict. For example, the studies with a small sample size were excluded to minimize the risk of selection bias. Fourth, the included clinical trials were not only ASI studies but also PSI studies, and the latter part of studies might be more worthwhile because it was often ignored by previous evidence-based studies.

Our meta-analysis has also limitations that affect interpretation of the results. First, only 7 of the 25 studies that we included were randomized control trials. We collected data from the nonrandomized or historical cohorts, which may have affected the results. Selection issues in some of these trials were not very well explained. Thus, a selection bias leading to changes in results may not be excluded. Second, the number of patients (6 to 1062 patients) and the follow-up period (7 days to over 3 years) varied greatly among the clinical trials. Overall, most of the included studies had a relatively short follow-up duration, and large samples and multicenter clinical trials regarding specific immunotherapy for PC were insufficient. The variables may introduce some level of bias. For example, the heterogeneity in the data shown in Figures 2 and 5 was significant. Thus, implementation and distribution biases, which might influence the reliability, might exist in the results of meta-analysis. Third, only the mean and standard deviations could be applied to our meta-analysis for the continuous data. However, the data that were expressed by the median and the range in some papers were excluded from the meta-analysis. Fourth, the included studies lacked sufficient patient information, such as adverse events, and negative trial outcomes were often not published. Thus, our data analysis might cause an overestimation of the immunotherapy effects. Fifth, the number or dose of specific immunotherapy cells, antibodies, and vaccines that were infused was variable among different studies, and further research should compare the clinical outcomes of PC-specific immunotherapy among patients receiving variable number or dose of cells, antibodies, and vaccines. In most of the studies, the specific immunotherapy was infused via injection. We did not stratify the results according to the types of PC-specific immunotherapy and routes of the immunotherapy infusion. Therefore, further work might be necessary to compare the cost-effectiveness of different types of PC-specific immunotherapy and routes of certain immunotherapy.

Our analysis collectively demonstrated that specific immunotherapy can result in prolonged OS in PC patients. We also found that these specific immunotherapy-mediated improvements typically correspond to enhanced immunity function and serum cancer marker inhibition. Hence, the efficacy of specific immunotherapy in the area of clinical outcomes is attributed to its possible application as a promising therapy for PC. However, this immunotherapy should be further developed.

## Supplementary Material

Supplementary materials included 8 Supplementary Tables. Supplementary Table 1 and 2 descripted the Quality of studies with self-control data and studies with case-control data. Supplementary Table 3 and 4 descripted the subgroup results of meta-analyses for 3-month to 3-year OS. Supplementary Table 5 descripted the subgroup results of meta-analyses about immune responses before and after specific immunotherapy. Supplementary Table 6 descripted the subgroup results of meta-analyses about lymphocyte subset percentages. Supplementary Table 7 and 8 descripted the subgroup results of meta-analyses about cytokines and serum cancer markers before and after specific immunotherapy.

## Figures and Tables

**Table 1:** Clinical information from eligible trials used in the meta-analysis.

Author, journal (year) [Ref]	Country	Age	Sex (M/F)	Number of pts. (case/control)	Study design	Enrollment period	Tumor stage	Specific immunotherapy arm	Control arm	Specific immunotherapy course	Length of follow-up
Asahara et al., J Transl Med (2013) [8]	Japan	Median (range) 61.3 (33–80) years	66/46	112 (31/81)	Retrospective case-control study	2009.3–2010.2	Unknown	KIF20A-66 HLA-A24-restricted peptide + chemotherapy	Chemotherapy	1.0 or 3.0 mg/body peptide × 8 in a 56 days cycle + 1.0 or 3.0 mg/m^2^ gemcitabine per 2 weeks	Unknown
Bernhardt et al., Br J Cancer (2006) [9]	Norway	Range: 40–72 years	26/22	48 (26/22)	Phase I/II case-control study	2000.9–2003.3	Unknown	Telomerase peptide vaccine	Negative peptide	3 injections in week 1 and 1 weekly injection in weeks 2, 3, 4, 6, and 10	10–575 days (survival days from start of treatment)
Brett et al., J Clin Oncol (2002) [10]	UK	Median (range) 62.95 (44–79) years	24/6	30	Phase II self-control study	Unknown	II, III, IV	Anti-gastrin-17 antibody	Unknown	3 doses of 100 *μ*g or 250 *μ*g of G17DT on weeks 0, 2, and 6	16 weeks
Cai et al., Chinese Journal of Cancer Biotherapy (2013) [11]	China	Median (range) 64 (30–75) years	13/11	24	Self-control study	2011.7–2012.5	III, IV	DC pulsed with antigens of PANC-1 cells + CIK	Unknown	1 × 10^6^ DC × 4 + 1 × 10^6^ CIK × 3	4 weeks after first treatment to 16.7 months
Chawla et al., Mol Ther (2010) [12]	USA	Median (range) 64 (50–83) years	7/6	13 (7/6)	Phase I/II case-control study	2007.10–2009.1	Unknown	Positive Rexin-G vaccine	Negative vaccine	1-2 × 10^11^ cfu Rexin-G × 12	0–12 months
Endo et al., J Hepatobiliary Pancreat Sci (2012) [13]	Japan	Mean age 62 ± 8.1 years	13/11	24 (9/15)	Phase I case-control study	2003.11–2007.7	IA, IIA, IIB, IV	DC + OK432	OK432	1.0 × 10^8^ DC + 0.1 mL OK432	0.7–6.1 years
Gjertsen et al., Int J Cancer (2001) [14]	Norway	Range: 35–77 years	19/18	37 (17/20)	Phase I/II case-control study	1996.11–1998.11	Unknown	K-ras peptide vaccination + GM-CSF	Negative peptide	100 *μ*g (single mutant ras peptide or a mixture of 4 mutant ras peptides) × 4 + 40 *μ*g GM-CSF × 4	3–6 months after first injection to 2 years
Kameshima et al., Cancer Sci (2013) [15]	Japan	Range 50–80 years	3/3	6	Self-control study	2005.12–2010.11	Unknown	Survivin-2B80-88 peptide + IFA + *α*-interferon	Unknown	(1 mg/1 mL peptide + 1 mL IFA) × 4 + 3,000 IU *α*-interferon × 12	Unknown
Koido et al., Clin Cancer Res (2014) [16]	Japan	Range 39–73 years	7/4	11	Phase I self-control study	2011.8–2013.1	IV	DC pulsed with multiple WT-1 peptides + chemotherapy	Unknown	1 × 10^7^ DC/WT-1 cells/dose × 5 + 1000 mg/m^2^ gemcitabine × 5	800 days after first injection
Kondo et al., Anticancer Res (2008) [17]	Japan	Range 51–84 years	14/6	20	Self-control study	2001–2006	III, IV	DCs pulsed with MUC1 peptide + CTLs stimulated by MUC1-expressing cells	Unknown	(1.1 × 10^7^ to 3.1 × 10^8^ MUC1-DCs + 5.0 × 10^8^ to 6.8 × 10^9^) × 2 to 15 times	Unknown
Kubuschok et al., Hum Gene Ther (2012) [18]	Germany	Range 48–66 years	4/3	7	Self-control study	1997–2000	II, III	muRas-LCL	Unknown	5 × 10^6^ muRas-LCL × 8	7–52 weeks
Le et al., J Immunother (2013) [19]	USA	Median (range) 62 (44–77) years	21/9	30 (15/15)	Phase Ib, randomized study	2009.3–2010.12	II, III, IV	Ipilimumab + GVAX	Ipilimumab	10 mg/kg ipilimumab + 2.5 × 10^8^ cells of GVAX	0–30 months
Le et al., J Clin Oncol (2015) [20]	USA	Median (range) 63 (45–87) years	53/37	90 (61/29)	Multicenter, randomized, case-control phase II trial	2011.9–2012-11	Unknown	Cy/GVAX plus CRS-207	Cy/GVAX	(200 mg/m^2^ cy + 2.5 × 10^8^ cells GVAX) × 6 + 1 × 10^9^ colony-forming units CRS-207 × 6	600 days after first treatment
Liu, Chin J Clinicians (2012) [21]	China	Median (range) 61 (42–76) years	27/23	50 (25/25)	Randomized, case-control study	2010.6–2011.12	I, II, III, IV	DC pulsed with tumor cells lysates + CIK + chemotherapy	Chemotherapy	2 × 10^9^ DC-CIK cells × 6 + 600 mg/m^2^ 5-FU	Unknown
Liu et al., Nanjing yi ke da xue xue bao (2010) [22]	China	Range 48–79 years	17/13	30 (15/15)	Randomized, case-control study	Unknown	III, IV	DC pulsed with tumor cells lysates + CIK + chemotherapy	Chemotherapy	DC × 4 + 1 g/m^2^ chemotherapy × 18	7 days after therapy
Middleton et al., Lancet Oncol (2014) [23]	UK	Range 55–69 years	608/454	1062 (704/358)	Multicenter, open-label, phase III randomized control trial	2007.3–2011.3	Unknown	Telomerase peptide vaccine GV1001 + gemcitabine and capecitabine	Gemcitabine, capecitabine	1000 mg/m^2^ gemcitabine × 6 + 830 mg/m^2^ capecitabine × 2 + GV1001	Followed up for a median of 6.0 months
Nishida et al., J Immunother (2014) [24]	Japan	Median (range) 60 (41–75) years	17/15	32	Phase I self-control study	2008–2010	Unknown	WT-1 peptides vaccine + gemcitabine	Unknown	1000 mg/m^2^ gemcitabine × 6 + 0.3 to 3.0 mg WT-1 vaccine × 4	30 months
Picozzi et al., Eur J Cancer (2015) [25]	USA	Range 39–80 years	33/25	58 (29/29)	Phase Ib case-control study	2102.6–2013.2	Unknown	^90^Y-clivatuzumab tetraxetan (anti-MUC5ac monoclonal antibody) + gemcitabine	^90^Y-clivatuzumab tetraxetan	6.5 mCi/m^2^ Y-clivatuzumab tetraxetan × 3 ± 200 mg/m^2^ gemcitabine × 4	12 months after first injection
Qiu et al., Int J Clin Oncol (2013) [26]	China	Mean (range) age 60.1 ± 8.4 (44–90) years	12/2	14	Phase I self-control study	2004.3–2009.2	III, IV	DC pulsed with *α*-Gal expressing cancer cell lysate + CIK	Unknown	(2 × 10^9^cells–10 × 10^9^cells) (DCs + CIKs)/injection × 1–5 times	24 months after first injection
Sultana et al., BMC Cancer (2009) [27]	UK	Median (range) 60 (47–67) years	Unknown	19	Randomized phase II self-control trial	2003.2–2005.7	IVa, IVb	Anti-carcinoembryonic antigen I^131^KAb201 antibodies	Unknown	50 mCi–75 mCi KAb 201 via either the intra-arterial or intravenous delivery route	90 days posttreatment
Suzuki et al., J Immunother (2014) [28]	Japan	Median (range) 62 (48–74) years	4/5	9	Nonrandomized, open-label, phase I self-control study	Unknown	III, IV	KIF20A-10-66 peptide + gemcitabine	Unknown	0.5–3 mg/body KIF20A-10-66 peptide × 4 + 1000 mg/m^2^ gemcitabine × 3	94–366 days
Wen et al., Xiandai zhongxiyi jiehe zazhi (2013) [29]	China	Range 49–78 years	33/27	60 (30/30)	Randomized, case-control study	2008.3–2011.3	Unknown	DC pulsed with tumor cells lysates + CIK + chemotherapy	Chemotherapy	2 × 10^9^ DC-CIK cells × 6 + 600 mg/m^2^ 5-FU	2–26 months
Yanagimoto et al., Oncol Rep (2010) [30]	Japan	Median (range) 64 (48–80) years	13/8	21	Nonrandomized, open-label, phase II self-control study	2006.9–2008.3	IVa, IVb	PPV + gemcitabine	Unknown	3 mg/peptide were administered weekly + 1000 mg/m^2^ gemcitabine per week for 3 weeks	3–24 months
Yutani et al., Oncol Rep (2013) [31]	Japan	Median (range) 61 (44–78) years	27/14	41	Open-label phase II self-control study	2008.11–2011.3	IVa, IVb	PPV	Unknown	4 peptides (3 mg/each peptide) administered once a week for 6 consecutive weeks	Over 800 days
Zhang, Zhongguo xian dai yi xue za zhi (2014) [32]	China	Mean range 58.2 ± 5.2 (48–78) years	33/27	60 (30/30)	Randomized, case-control study	2010.1–2012.6	I, II, III, IV	DC pulsed with tumor cells lysates + CIK + chemotherapy	Chemotherapy	2 × 10^9^ DC-CIK cells × 4 + 15 mg/kg 5-FU × 4	Over 3 years

DC, dendritic cell; CIK, cytokine-induced killer cell; GM-CSF, granulocyte-macrophage colony-stimulating factor; IFA, incomplete Freund's adjuvant; WT-1, Wilms' tumor-1; MUC1, Mucin 1; LCL; lymphoblastoid cell lines; GVAX, GM-CSF cell-based vaccines; *α*-Gal, alpha-galactosyl; PPV, personalized peptide vaccination; CRS-207, human mesothelin; ipilimumab, anti-CTLA-4 antibody.
