# Supplementary material for: Clinical Outcomes of Specific Immunotherapy in Advanced Pancreatic Cancer: A Systematic Review and Meta-Analysis
Source: J Immunol Res. 2017 Feb 7;2017:8282391. doi: 10.1155/2017/8282391 (PMC5318641; doi:10.1155/2017/8282391)
Supplement: Supplementary file 1 — Supplementary materials included 8 Supplementary Tables. Supplementary Table 1 and 2 descripted the Quality of studies with self-control data and studies with case-control data. Supplementary Table 3 and 4 descripted the subgroup results of meta-analyses for 3-month to 3-year OS. Supplementary Table 5 descripted the subgroup results of meta-analyses about immune responses before and after specific immunotherapy. Supplementary Table 6 descripted the subgroup results of meta-analyses about lymphocyte subset percentages. Supplementary Table 7 and 8 descripted the subgroup results of meta-analyses about cytokines and serum cancer markers before and after specific immunotherapy. [file 8282391.f1.pdf]

Supplementary Tables

Supplementary Table 1. Quality of studies with self-control data

Author (Year)	Q1	Q2	Q3	Q4	Q5	Q6	Q7	Quality
Brett (2002)	Yes	No	Yes	Yes	Yes	Yes	Yes	High
Cai (2013)	Yes	Yes	Yes	Yes	Yes	Yes	Yes	High
Kameshima (2013)	Yes	Yes	Yes	Yes	No	Yes	No	Low
Koido (2014)	Yes	Yes	Yes	Yes	Yes	Yes	Yes	High
Kondo (2008)	Unclear	Yes	Yes	Yes	Yes	Yes	No	Low
Kubuschok (2012)	No	Yes	Yes	Yes	Yes	Yes	Yes	High
Nishida (2014)	Yes	Yes	Yes	Yes	No	Yes	Yes	High
Qiu (2013)	Unclear	Yes	Yes	Yes	Yes	Yes	Yes	High
Sultana (2009)	Unclear	Yes	Yes	No	Yes	No	Yes	Low
Suzuki (2014)	Yes	No	Yes	Yes	Yes	Yes	Yes	High
Yanagimoto (2010)	Yes	Yes	No	Yes	Yes	Yes	Yes	High
Yutani (2013)	Yes	Yes	No	Yes	Yes	Yes	Yes	High

Supplementary Table 2. Quality of studies with case-control data

Author (Year)	Q1	Q2	Q3	Q4	Q5	Q6	Q7	Quality
Asahara (2013)	No	No	No	Yes	Yes	Yes	No	Low
Bernhardt (2006)	Yes	Yes	No	Yes	Yes	Yes	No	Low
Chawla (2010)	Yes	No	No	Yes	Yes	Yes	Yes	Low
Endo (2012)	Yes	Yes	No	Yes	Yes	Yes	Yes	High
Gjertsen (2001)	Yes	Yes	No	Yes	Yes	Yes	Yes	High
Le (2013)	Yes	Yes	Yes	Yes	Yes	Yes	Yes	High
Le (2015)	Yes	Yes	Yes	Yes	Yes	Yes	Yes	High
Liu (2012)	Unclear	Yes	No	Yes	Yes	Yes	No	Low
Liu (2010)	Unclear	Yes	No	Yes	Yes	Yes	Yes	Low
Middleton (2014)	Yes	Yes	Yes	Yes	Yes	Yes	Yes	High
Picozzi (2015)	Yes	No	No	Yes	Yes	Yes	Yes	Low
Wen (2013)	Yes	Yes	Yes	Yes	Yes	Yes	No	High
Zhang (2012)	Unclear	No	No	Yes	Yes	Yes	Yes	Low

Supplementary Table 3. Subgroup results of meta-analyses about 3-month to 12-month OS

Study Group	Description	No. of studies	OR (95% CI)	Significance *P* Value	I^2 (%)	Heterogeneity *P* Value
**3-month OS**						
Therapy Type	ASI	4	6.39 (1.07-38.16)	0.04	73	0.006
	PSI	2	1.96 (0.74-5.19)	0.17	0	0.63
Tumor Stage	IV Grade Alone	1	3.43 (0.29-40.95)	0.33	0	>0.99
	Mixed or Unknown	5	4.54 (1.27-16.31)	0.02	68	0.008
Study Quality	High	3	3.79 (0.65-22.19)	0.14	64	0.04
	Low	3	5.66 (0.75-42.79)	0.09	72	0.03
Study Location	America	2	1.55 (0.76-3.18)	0.23	0	0.73
	Asia	1	0.57 (0.03-10.43)	0.71	0	>0.99
	Europe	3	16.94 (3.97-72.24)	0.0001	17	0.3
**6-month OS**						
Therapy Type	ASI	7	3.70 (1.22-11.24)	0.02	72	0.001
	PSI	5	3.31 (1.63-6.74)	0.001	0	0.9
Tumor Stage	III & IV Grade	2	2.76 (0.72-10.58)	0.14	0	0.54
	IV Grade Alone	2	3.28 (0.57-18.97)	0.18	13	0.28
	Mixed or Unknown	8	3.44 (1.39-8.49)	0.007	73	0.0005
Study Quality	High	9	3.38 (1.41-8.11)	0.006	68	0.002
	Low	3	3.45 (1.32-9.05)	0.01	0	0.7
Study Location	America	5	3.27 (1.72-6.21)	0.003	0	0.9
	Asia	3	4.63 (0.80-26.74)	0.09	30	0.24
	Europe	4	3.64 (0.79-16.78)	0.1	76	0.006
**12-month OS**						
Therapy Type	ASI	10	3.05 (1.49-6.24)	0.002	53	0.02
	PSI	4	5.42 (1.39-21.18)	0.02	0	0.92
Tumor Stage	III & IV Grade	2	4.62 (0.84-25.43)	0.08	0	0.56
	IV Grade Alone	2	11.51 (1.61-82.82)	0.02	0	0.68
	Mixed or Unknown	10	1.59 (1.18-2.13)	0.002	46	0.05
Study Quality	High	9	1.53 (1.13-2.06)	0.005	52	0.03
	Low	5	5.23 (1.96-13.93)	0.0009	0	0.98
Study Location	America	4	5.00 (1.64-15.24)	0.005	0	0.96
	Asia	5	3.50 (1.51-8.10)	0.003	26	0.25
	Europe	5	2.15 (0.77-5.98)	0.14	26	0.25

Supplementary Table 4. Subgroup results of meta-analyses about 1.5-year to 3-year OS						
**Study Group**	**Description**	**No. of studies**	**OR (95% CI)**	**Significance *P* Value**	**I^2 (%)**	**Heterogeneity *P* Value**
**1.5-year OS**						
Therapy Type	ASI	7	1.23 (0.77-1.97)	0.39	0	0.48
	PSI	2	2.73 (0.45-16.67)	0.28	0	0.56
Tumor Stage	III & IV Grade	2	2.73 (0.45-16.67)	0.28	0	0.56
	IV Grade Alone	1	7.93 (0.35-179.96)	0.19	0	>0.99
	Mixed or Unknown	6	1.18 (0.73-1.90)	0.5	0	0.54
Study Quality	High	8	1.28 (0.80-2.02)	0.3	0	0.49
	Low	1	2.65 (0.1-68.3)	0.56	0	>0.99
Study Location	America	2	2.97 (0.31-28.01)	0.34	0	0.55
	Asia	3	2.97 (0.59-14.88)	0.18	24	0.27
	Europe	4	1.13 (0.69-1.86)	0.62	0	0.6
**2-year OS**						
Therapy Type	ASI	3	1.57 (0.41-6.01)	0.51	0	0.51
	PSI	3	8.84 (1.46-53.52)	0.02	0	0.84
Tumor Stage	III & IV Grade	2	10.4 (1.12-96.68)	0.04	0	0.59
	IV Grade Alone	2	1.13 (0.26-5.01)	0.87	0	0.57
	Mixed or Unknown	2	6.56 (0.74-57.97)	0.09	0	0.99
Study Quality	High	6	2.91 (0.99-8.53)	0.05	0	0.55
Study Location	America	1	5.74 (0.25-130.37)	0.27	0	>0.99
	Asia	3	1.58 (0.42-6.03)	0.5	0	0.5
Study Location	America	1	5.74 (0.25-130.37)	0.27	0	>0.99
	Asia	3	1.58 (0.42-6.03)	0.5	0	0.5
	Europe	2	11.14 (1.21-103.04)	0.03	0	0.64
**3-year OS**						
Therapy Type	ASI	3	2.74 (1.10-6.86)	0.03	0	0.92
	PSI	1	3.46 (0.12-100.51)	0.47	0	>0.99
Tumor Stage	III & IV Grade	1	3.46 (0.12-100.51)	0.47	0	>0.99
	Mixed or Unknown	3	2.74 (1.10-6.86)	0.03	0	0.92
Study Quality	High	3	2.28 (0.46-11.20)	0.31	0	0.96
	Low	1	3.05 (1.05-8.84)	0.04	0	>0.99
Study Location	Asia	3	2.74 (1.10-6.86)	0.03	0	0.92
	Europe	1	3.46 (0.12-100.51)	0.47	0	>0.99

Supplementary Table 5. Subgroup results of meta-analyses about immune responses before and after specific immunotherapy						
**Study Group**	**Description**	**No. of studies**	**OR (95% CI)**	**Significance *P* Value**	**I^2 (%)**	**Heterogeneity *P* Value**
**CTL-response**						
Therapy Type	ASI	3	3.63 (1.72-7.65)	0.0007	0	0.97
Tumor Stage	III & IV Grade	1	4.0 (0.56-28.40)	0.17	0	>0.99
	IV Grade Alone	1	4.0 (1.11-14.43)	0.03	0	>0.99
	Mixed or Unknown	1	3.31 (1.17-9.36)	0.02	0	>0.99
Study Quality	High	2	4.0 (1.37-11.70)	0.01	0	1
	Low	1	3.31 (1.17-9.36)	0.02	0	>0.99
Study Location	Asia	3	3.63 (1.72-7.65)	0.0007	0	0.97
**Antibody-response**						
Therapy Type	ASI	2	2.94 (1.37-6.29)	0.006	0	0.83
	PSI	1	3.45 (1.19-9.99)	0.02	0	>0.99
Tumor Stage	III & IV Grade	1	3.45 (1.19-9.99)	0.02	0	>0.99
	IV Grade Alone	2	2.94 (1.37-6.29)	0.006	0	0.83
Study Quality	High	3	3.10 (1.67-5.76)	0.0003	0	0.95
Study Location	Asia	2	2.94 (1.37-6.29)	0.006	0	0.83
	Europe	1	3.45 (1.19-9.99)	0.02	0	>0.99

Supplementary Table 6. Subgroup results of meta-analyses about lymphocyte subset percentages						
**Study Group**	**Description**	**No. of studies**	**OR (95% CI)**	**Significance *P* Value**	**I^2 (%)**	**Heterogeneity *P* Value**
**CD4+**						
Therapy Type	ASI	4	7.89 (0.30-15.48)	0.04	97	<0.00001
Tumor Stage	III & IV Grade	2	7.58 (-10.64-25.81)	0.41	98	<0.00001
	Mixed or Unknown	2	7.81 (1.31-14.32)	0.02	92	0.0006
Study Quality	High	1	-1.90 (-7.23-3.43)	0.48	0	>0.99
	Low	3	10.85 (3.02-18.69)	0.007	97	<0.00001
Study Location	Asia	4	7.89 (0.30-15.48)	0.04	97	<0.00001
**CD8+**						
Therapy Type	ASI	4	7.89 (0.30-15.48)	0.04	97	<0.00001
Tumor Stage	III & IV Grade	2	7.58 (-10.64-25.81)	0.41	98	<0.00001
	Mixed or Unknown	2	7.81 (1.31-14.32)	0.02	92	0.0006
Study Quality	High	1	-1.90 (-7.23-3.43)	0.48	0	>0.99
	Low	3	10.85 (3.02-18.69)	0.007	97	<0.00001
Study Location	Asia	4	7.89 (0.30-15.48)	0.04	97	<0.00001
**CD56+**						
Therapy Type	ASI	6	3.74 (-2.46-9.94)	0.24	99	<0.00001
Tumor Stage	III & IV Grade	4	3.15 (-5.71-12.00)	0.49	99	<0.00001
	Mixed or Unknown	2	5.05 (0.74-9.36)	0.02	0	>0.99
Study Quality	High	2	6.68 (-3.99-17.36)	0.22	91	0.0009
	Low	4	2.37 (-5.39-10.12)	0.55	99	<0.00001
Study Location	Asia	6	3.74 (-2.46-9.94)	0.24	99	<0.00001
**CD4+/CD8+**						
Therapy Type	ASI	4	0.38 (0.31-0.44)	<0.0001	0	0.61
Tumor Stage	III & IV Grade	2	0.51 (0.27-0.76)	<0.0001	0	0.56
	Mixed or Unknown	2	0.36 (0.29-0.43)	<0.0001	0	0.68
Study Quality	High	1	0.40 (-0.06-0.86)	0.09	0	>0.99
	Low	3	0.37 (0.31-0.44)	<0.0001	0	0.4
Study Location	Asia	4	0.38 (0.31-0.44)	<0.0001	0	0.61
**CD4+CD25+**						
Therapy Type	ASI	3	-2.66 (-4.35 to -0.96)	0.002	79	0.009
Tumor Stage	III & IV Grade	1	-1.20 (-2.22 to -0.18)	0.02	0	>0.99
	Mixed or Unknown	2	-3.52 (-4.16 to -2.44)	<0.00001	0	0.85
Study Quality	High	1	-1.20 (-2.22 to -0.18)	0.02	0	>0.99
	Low	2	-3.52 (-4.16 to -2.44)	<0.00001	0	0.85
Study Location	Asia	3	2.66 (-4.35 to -0.96)	0.002	79	0.009

Supplementary Table 7. Subgroup results of meta-analyses about cytokine before and after specific immunotherapy						
**Study Group**	**Description**	**No. of studies**	**OR (95% CI)**	**Significance *P* Value**	**I^2 (%)**	**Hete**
**IFN-γ**						
Therapy Type	ASI	4	3.75 (0.77-6.73)	0.01	77	
Tumor Stage	III & IV Grade	1	19.13 (10.03-28.23)	<0.0001	0	
	IV Grade Alone	1	2.22 (-25.41-29.85)	0.87	0	
	Mixed or Unknown	2	2.28 (1.33-3.22)	<0.00001	0	
Study Quality	High	1	2.22 (-25.41-29.85)	0.87	0	
	Low	3	3.87 (0.75-7.00)	0.02	85	
Study Location	Asia	4	3.75 (0.77-6.73)	0.01	77	

Supplementary Table 8. Subgroup results of meta-analyses about serum cancer marker before and after specific immunotherapy						
**Study Group**	**Description**	**No. of studies**	**OR (95% CI)**	**Significance *P* Value**	**I^2 (%)**	**Heterogeneity *P* Value**
**CA19-9**						
Therapy Type	ASI	4	-238.52 (-319.87 to -157.17)	<0.00001	61	0.05
Tumor Stage	III & IV Grade	2	-175.95 (-253.33 to -98.56)	<0.00001	0	0. 57
	Mixed or Unknown	2	-338.93 (-556.43 to -121.42)	0.002	60	0. 12
Study Quality	High	3	-223.65 (-296.91 to -150.40)	<0.00001	60	0. 08
	Low	1	-507.80 (-807.51 to -208.09)	0.0009	0	>0. 99
Study Location	Asia	3	-229.57 (-363.35 to -95.80)	0.0008	58	0. 09
	Europe	1	-266.00 (-292.60 to -239.40)	<0.00001	0	>0. 99
